# Supplementary material for: Refining the use of seed balls to support seagrass restoration
Source: PLoS One. 2026 Aug 17;21(8):e0351822. doi: 10.1371/journal.pone.0351822 (PMC13480609; doi:10.1371/journal.pone.0351822)
Supplement: Supplementary file 2 [file pone.0351822.s003.docx]

**Reviewer #1:** *The study is providing further evidence for the purposes of seagrass restoration using seeds, for improving methods and success of deployment and germination. The data is important for practitioners and the wider topic and may help improve future restoration trials.*

**Author Response:** We thank the reviewer for their positive assessment of the value and applicability of our research.

**Reviewer #1:** *The few concerns I have are with the experimental design. There seems numerous factors being tested, clay type, ratio of materials used, buried not buried and drying. This makes the study a little hard to follow when the methods and the results are described. I would like to see a simple diagram of the treatments used that could help clear this up. Also more justification in the methods for why the different ratios were then chosen. Why were only two of the treatments used for burial/non-burial? The diagram and improved tables of treatments could clarify this.*

**Author Response:** We completely agree that the experimental design required clearer articulation. To address this, we have completely overhauled **Table 1** so that it now serves as a clear, comprehensive matrix detailing the composition ratio, planting method, and specific drying condition for each of the 7 treatments. We also added a "Matrix Description / Purpose" column to explicitly state the justification for each treatment's inclusion.

In the text of the Methods section, we have added further justification for the ratio choices, noting that they were selected to test various permutations of binding strength and nutritional profiles based on our preliminary trials. Regarding the burial treatments, we have added text explaining that burial was strictly limited to the "naked seed" and basic "fireclay" treatments in order to establish the baseline maximum emergence potential for an unencased seed versus the most basic clay formulation, thereby preserving experimental space to test the viability of above-ground broadcast deployment for the more complex matrices.

**Reviewer #1:** *In tables 1 and 2, the treatment labels mention '3h dried' after the 1:4:1 ratio seed ball, but the treatments in the methods talk about 1 hour oven dried at 30 degrees for one drying treatment or 6 hours air-dried at room temperature. So what is the 3h dried? This needs explaining.*

**Author Response:** We apologize for this discrepancy, which was a typographical error in the original manuscript text. The air-drying treatment was indeed conducted for 3 hours, not 6. We have corrected the text in the "Experimental seed ball production" section of the Methods to accurately state: *"In the first drying treatment, seed balls (containing the seeds) were air-dried at room temperature for three hours (labelled '3h dried')..."* to ensure total consistency with Tables 1 and 2.

**Reviewer #1:** *Also it seems this ration 1:4:1 ratio has been preferred for trialling the drying experiment. Why was this? Also why was the plain fireclay not used for any above ground experiments? There doesn't seem to be any balance.*

**Author Response:** These are excellent points that required clarification.

1. **Regarding the 1:4:1 ratio:** We have added a sentence to the Methods section explaining that the 1:4:1 formulation was selected for the drying experiments because our preliminary tests established that it offered the most stable baseline balance of workability and structural integrity.
2. **Regarding plain fireclay:** We have clarified in the Methods that plain fireclay was not utilized for the above-ground (surface) experiments because preliminary observations indicated it completely lacked the cohesive strength to resist rapid degradation without the addition of bentonite or loam when exposed to unconfined aquatic conditions.

**Reviewer #1:** *If there is a workflow that leads to these stages I think it could be made clearer.*

**Author Response:** To improve the flow and clarity of the experimental design, we have added a brief overview paragraph at the very beginning of the Methods section outlining the three main stages of the workflow: 1) material selection and preparation, 2) preliminary trials to assess clay structural integrity and define optimal ratios, and 3) the primary mesocosm experiment evaluating biological performance.

**Reviewer #1:** *The data is useful so I think it should be published so that more work can be done on it, but I think the submission can be improved.*

**Author Response:** We appreciate the reviewer's encouragement and believe the suggested revisions have significantly strengthened the clarity and quality of the manuscript.

**Reviewer #1:** *The 'naked seedball' treatment, is this just seeds? So not in any sort of ball? I would keep the wording consistent throughout so that readers are clear on this.*

**Author Response:** Yes, this treatment consisted entirely of unencased seeds to serve as our experimental control. We have revised the text in the Methods section to explicitly define this: *"Seed balls from the ‘naked seed’ treatment (which consisted solely of unencased seeds, not formulated into any sort of ball, acting as an experimental control)..."* We have also reviewed the entire manuscript to ensure the terminology remains strictly as "naked seeds" rather than "naked seedballs" for absolute clarity.

**Reviewer #1:** *I have added some minor annotations on the attached pdf, but mostly my comments are addressed here.*

**Author Response:** Thank you. We have reviewed the PDF annotations and ensured that any remaining minor typographical or formatting corrections have been incorporated into the revised manuscript file.
