# Supplementary material for: Refining the use of seed balls to support seagrass restoration
Source: PLoS One. 2026 Aug 17;21(8):e0351822. doi: 10.1371/journal.pone.0351822 (PMC13480609; doi:10.1371/journal.pone.0351822)
Supplement: Peer review history

# Peer review: Decision Letter 0

**DOI:** 10.1371/journal.pone.0351822.r001

**Authors:** Amitava Mukherjee

2 Jul 2026

REFINING THE USE OF SEED BALLS TO SUPPORT SEAGRASS RESTORATION

PLOS One

Dear Dr. Unsworth,

Thank you for submitting your manuscript to PLOS ONE. After careful consideration, we feel that it has merit but does not fully meet PLOS ONE’s publication criteria as it currently stands. Therefore, we invite you to submit a revised version of the manuscript that addresses the points raised during the review process.

Please submit your revised manuscript by Aug 01 2026 11:59PM. If you will need more time than this to complete your revisions, please reply to this message or contact the journal office at plosone@plos.org. When you're ready to submit your revision, log on to https://www.editorialmanager.com/pone/ and select the 'Submissions Needing Revision' folder to locate your manuscript file.

Please include the following items when submitting your revised manuscript:

- A letter that responds to each point raised by the academic editor and reviewer(s). You should upload this letter as a separate file labeled 'Response to Reviewers'.
- A marked-up copy of your manuscript that highlights changes made to the original version. You should upload this as a separate file labeled 'Revised Manuscript with Track Changes'.
- An unmarked version of your revised paper without tracked changes. You should upload this as a separate file labeled 'Manuscript'.

If you would like to make changes to your financial disclosure, please include your updated statement in your cover letter. Guidelines for resubmitting your figure files are available below the reviewer comments at the end of this letter.

If applicable, we recommend that you deposit your laboratory protocols in protocols.io to enhance the reproducibility of your results. Protocols.io assigns your protocol its own identifier (DOI) so that it can be cited independently in the future. For instructions see: https://journals.plos.org/plosone/s/submission-guidelines#loc-laboratory-protocols. Additionally, PLOS ONE offers an option for publishing peer-reviewed Lab Protocol articles, which describe protocols hosted on protocols.io. Read more information on sharing protocols at https://plos.org/protocols?utm_medium=editorial-email&utm_source=authorletters&utm_campaign=protocols.

As the corresponding author, your ORCID iD is verified in the submission system and will appear in the published article. PLOS supports the use of ORCID, and we encourage all coauthors to register for an ORCID iD and use it as well. Please encourage your coauthors to verify their ORCID iD within the submission system before final acceptance, as unverified ORCID iDs will not appear in the published article. *Only*  the individual author can complete the verification step; PLOS staff *cannot*  verify ORCID iDs on behalf of authors.

We look forward to receiving your revised manuscript.

Kind regards,

Amitava Mukherjee, ME, Ph.D.

Academic Editor

PLOS One

Journal Requirements:

When submitting your revision, we need you to address these additional requirements.

1.Please ensure that your manuscript meets PLOS ONE's style requirements, including those for file naming. The PLOS ONE style templates can be found at

https://journals.plos.org/plosone/s/file?id=wjVg/PLOSOne_formatting_sample_main_body.pdf and

https://journals.plos.org/plosone/s/file?id=ba62/PLOSOne_formatting_sample_title_authors_affiliations.pdf

2. In your Methods section, please provide additional information regarding the permits you obtained for the work. Please ensure you have included the full name of the authority that approved the field site access and, if no permits were required, a brief statement explaining why.

3. We note that the grant information you provided in the ‘Funding Information’ and ‘Financial Disclosure’ sections do not match.

When you resubmit, please ensure that you provide the correct grant numbers for the awards you received for your study in the ‘Funding Information’ section.

4. Thank you for stating the following financial disclosure:

“Heritage Lottery Fund”

Please state what role the funders took in the study.  If the funders had no role, please state: "The funders had no role in study design, data collection and analysis, decision to publish, or preparation of the manuscript."

If this statement is not correct you must amend it as needed.

Please include this amended Role of Funder statement in your cover letter; we will change the online submission form on your behalf.

5. Thank you for stating the following in the Competing Interests section:

“The authors have declared that no competing interests exist.”

We note that one or more of the authors are employed by a commercial company: Independent Researcher, Brackla Industrial Estate,  and The Fieldwork Company

1.        Please provide an amended Funding Statement declaring this commercial affiliation, as well as a statement regarding the Role of Funders in your study. If the funding organization did not play a role in the study design, data collection and analysis, decision to publish, or preparation of the manuscript and only provided financial support in the form of authors' salaries and/or research materials, please review your statements relating to the author contributions, and ensure you have specifically and accurately indicated the role(s) that these authors had in your study. You can update author roles in the Author Contributions section of the online submission form.

Please also include the following statement within your amended Funding Statement.

“The funder provided support in the form of salaries for authors [insert relevant initials], but did not have any additional role in the study design, data collection and analysis, decision to publish, or preparation of the manuscript. The specific roles of these authors are articulated in the ‘author contributions’ section.”

If your commercial affiliation did play a role in your study, please state and explain this role within your updated Funding Statement.

2. Please also provide an updated Competing Interests Statement declaring this commercial affiliation along with any other relevant declarations relating to employment, consultancy, patents, products in development, or marketed products, etc.

Within your Competing Interests Statement, please confirm that this commercial affiliation does not alter your adherence to all PLOS ONE policies on sharing data and materials by including the following statement: "This does not alter our adherence to  PLOS ONE policies on sharing data and materials.” (as detailed online in our guide for authors http://journals.plos.org/plosone/s/competing-interests) . If this adherence statement is not accurate and  there are restrictions on sharing of data and/or materials, please state these. Please note that we cannot proceed with consideration of your article until this information has been declared.

Please include both an updated Funding Statement and Competing Interests Statement in your cover letter. We will change the online submission form on your behalf.

6. If the reviewer comments include a recommendation to cite specific previously published works, please review and evaluate these publications to determine whether they are relevant and should be cited. There is no requirement to cite these works unless the editor has indicated otherwise.

7. Please review your reference list to ensure that it is complete and correct. If you have cited papers that have been retracted, please include the rationale for doing so in the manuscript text, or remove these references and replace them with relevant current references. Any changes to the reference list should be mentioned in the rebuttal letter that accompanies your revised manuscript. If you need to cite a retracted article, indicate the article’s retracted status in the References list and also include a citation and full reference for the retraction notice.

[Note: HTML markup is below. Please do not edit.]

Reviewers' comments:

Reviewer's Responses to Questions

**Comments to the Author**

1. Is the manuscript technically sound, and do the data support the conclusions?

Reviewer #1: Partly

**********

2. Has the statistical analysis been performed appropriately and rigorously?

Reviewer #1: Yes

**********

3. Have the authors made all data underlying the findings in their manuscript fully available?

The PLOS Data policy

Reviewer #1: Yes

**********

4. Is the manuscript presented in an intelligible fashion and written in standard English?

Reviewer #1: Yes

**********

Reviewer #1: The study is providing further evidence for the purposes of seagrass restoration using seeds, for improving methods and success of deployment and germination. The data is important for practitioners and the wider topic and may help improve future restoration trials.

The few concerns I have are with the experimental design. There seems numerous factors being tested, clay type, ratio of materials used, buried not buried and drying. This makes the study a little hard to follow when the methods and the results are described. I would like to see a simple diagram of the treatments used that could help clear this up. Also more justification in the methods for why the different ratios were then chosen. Why were only two of the treatments used for burial/non-burial? The diagram and improved tables of treatments could clarify this.

In tables 1 and 2, the treatment labels mention '3h dried' after the 1:4:1 ratio seed ball, but the treatments in the methods talk about 1 hour oven dried at 30 degrees for one drying treatment or 6 hours air-dried at room temperature. So what is the 3h dried? This needs explaining.

Also it seems this ration 1:4:1 ratio has been preferred for trialling the drying experiment. Why was this? Also why was the plain fireclay not used for any above ground experiments? There doesn't seem to be any balance.

If there is a workflow that leads to these stages I think it could be made clearer.

The data is useful so I think it should be published so that more work can be done on it, but I think the submission can be improved.

The 'naked seedball' treatment, is this just seeds? So not in any sort of ball? I would keep the wording consistent throughout so that readers are clear on this.

I have added some minor annotations on the attached pdf, but mostly my comments are addressed here.

**********

what does this mean?). If published, this will include your full peer review and any attached files.

If you choose “no”, your identity will remain anonymous but your review may still be made public.

**Do you want your identity to be public for this peer review?**  For information about this choice, including consent withdrawal, please see our Privacy Policy

Reviewer #1: No

**********

[NOTE: If reviewer comments were submitted as an attachment file, they will be attached to this email and accessible via the submission site. Please log into your account, locate the manuscript record, and check for the action link "View Attachments". If this link does not appear, there are no attachment files.]

To ensure your figures meet our technical requirements, please review our figure guidelines: https://journals.plos.org/plosone/s/figures

You may also use PLOS’s free figure tool, NAAS, to help you prepare publication quality figures: https://journals.plos.org/plosone/s/figures#loc-tools-for-figure-preparation.

NAAS will assess whether your figures meet our technical requirements by comparing each figure against our figure specifications.

Attachment Submitted filename: PONE-D-26-27071_reviewer.pdf

# Author comment: Author response to Decision Letter 1

**DOI:** 10.1371/journal.pone.0351822.r002

17 Jul 2026

Reviewer #1: The study is providing further evidence for the purposes of seagrass restoration using seeds, for improving methods and success of deployment and germination. The data is important for practitioners and the wider topic and may help improve future restoration trials.

Author Response: We thank the reviewer for their positive assessment of the value and applicability of our research.

Reviewer #1: The few concerns I have are with the experimental design. There seems numerous factors being tested, clay type, ratio of materials used, buried not buried and drying. This makes the study a little hard to follow when the methods and the results are described. I would like to see a simple diagram of the treatments used that could help clear this up. Also more justification in the methods for why the different ratios were then chosen. Why were only two of the treatments used for burial/non-burial? The diagram and improved tables of treatments could clarify this.

Author Response: We completely agree that the experimental design required clearer articulation. To address this, we have completely overhauled Table 1 so that it now serves as a clear, comprehensive matrix detailing the composition ratio, planting method, and specific drying condition for each of the 7 treatments. We also added a "Matrix Description / Purpose" column to explicitly state the justification for each treatment's inclusion.

In the text of the Methods section, we have added further justification for the ratio choices, noting that they were selected to test various permutations of binding strength and nutritional profiles based on our preliminary trials. Regarding the burial treatments, we have added text explaining that burial was strictly limited to the "naked seed" and basic "fireclay" treatments in order to establish the baseline maximum emergence potential for an unencased seed versus the most basic clay formulation, thereby preserving experimental space to test the viability of above-ground broadcast deployment for the more complex matrices.

Reviewer #1: In tables 1 and 2, the treatment labels mention '3h dried' after the 1:4:1 ratio seed ball, but the treatments in the methods talk about 1 hour oven dried at 30 degrees for one drying treatment or 6 hours air-dried at room temperature. So what is the 3h dried? This needs explaining.

Author Response: We apologize for this discrepancy, which was a typographical error in the original manuscript text. The air-drying treatment was indeed conducted for 3 hours, not 6. We have corrected the text in the "Experimental seed ball production" section of the Methods to accurately state: "In the first drying treatment, seed balls (containing the seeds) were air-dried at room temperature for three hours (labelled '3h dried')..." to ensure total consistency with Tables 1 and 2.

Reviewer #1: Also it seems this ration 1:4:1 ratio has been preferred for trialling the drying experiment. Why was this? Also why was the plain fireclay not used for any above ground experiments? There doesn't seem to be any balance.

Author Response: These are excellent points that required clarification.

1. Regarding the 1:4:1 ratio: We have added a sentence to the Methods section explaining that the 1:4:1 formulation was selected for the drying experiments because our preliminary tests established that it offered the most stable baseline balance of workability and structural integrity.

2. Regarding plain fireclay: We have clarified in the Methods that plain fireclay was not utilized for the above-ground (surface) experiments because preliminary observations indicated it completely lacked the cohesive strength to resist rapid degradation without the addition of bentonite or loam when exposed to unconfined aquatic conditions.

Reviewer #1: If there is a workflow that leads to these stages I think it could be made clearer.

Author Response: To improve the flow and clarity of the experimental design, we have added a brief overview paragraph at the very beginning of the Methods section outlining the three main stages of the workflow: 1) material selection and preparation, 2) preliminary trials to assess clay structural integrity and define optimal ratios, and 3) the primary mesocosm experiment evaluating biological performance.

Reviewer #1: The data is useful so I think it should be published so that more work can be done on it, but I think the submission can be improved.

Author Response: We appreciate the reviewer's encouragement and believe the suggested revisions have significantly strengthened the clarity and quality of the manuscript.

Reviewer #1: The 'naked seedball' treatment, is this just seeds? So not in any sort of ball? I would keep the wording consistent throughout so that readers are clear on this.

Author Response: Yes, this treatment consisted entirely of unencased seeds to serve as our experimental control. We have revised the text in the Methods section to explicitly define this: "Seed balls from the ‘naked seed’ treatment (which consisted solely of unencased seeds, not formulated into any sort of ball, acting as an experimental control)..." We have also reviewed the entire manuscript to ensure the terminology remains strictly as "naked seeds" rather than "naked seedballs" for absolute clarity.

Reviewer #1: I have added some minor annotations on the attached pdf, but mostly my comments are addressed here.

Author Response: Thank you. We have reviewed the PDF annotations and ensured that any remaining minor typographical or formatting corrections have been incorporated into the revised manuscript file.

Attachment Submitted filename: Reviewer.docx

# Peer review: Decision Letter 1

**DOI:** 10.1371/journal.pone.0351822.r003

**Authors:** Amitava Mukherjee

2 Aug 2026

REFINING THE USE OF SEED BALLS TO SUPPORT SEAGRASS RESTORATION

PONE-D-26-27071R1

Dear Dr. Unsworth,

We’re pleased to inform you that your manuscript has been judged scientifically suitable for publication and will be formally accepted for publication once it meets all outstanding technical requirements.

Within one week, you’ll receive an e-mail detailing the required amendments. When these have been addressed, you’ll receive a formal acceptance letter and your manuscript will be scheduled for publication.

An invoice will be generated when your article is formally accepted. Please note, if your institution has a publishing partnership with PLOS and your article meets the relevant criteria, all or part of your publication costs will be covered. Please make sure your user information is up-to-date by logging into Editorial Manager at Editorial Manager® and clicking the ‘Update My Information' link at the top of the page. For questions related to billing, please contact billing support.

If your institution or institutions have a press office, please notify them about your upcoming paper to help maximize its impact. If they’ll be preparing press materials, please inform our press team as soon as possible -- no later than 48 hours after receiving the formal acceptance. Your manuscript will remain under strict press embargo until 2 pm Eastern Time on the date of publication. For more information, please contact onepress@plos.org.

Kind regards,

Amitava Mukherjee, ME, Ph.D.

Academic Editor

PLOS One

Additional Editor Comments (optional):

Reviewers' comments:

Reviewer's Responses to Questions

**Comments to the Author**

Reviewer #1: All comments have been addressed

**********

2. Is the manuscript technically sound, and do the data support the conclusions?

Reviewer #1: Yes

**********

3. Has the statistical analysis been performed appropriately and rigorously?

Reviewer #1: Yes

**********

4. Have the authors made all data underlying the findings in their manuscript fully available?

The PLOS Data policy

Reviewer #1: Yes

**********

5. Is the manuscript presented in an intelligible fashion and written in standard English?

Reviewer #1: Yes

**********

Reviewer #1: Thank you for clarifying the points that I raised. The methods and experimental design make a lot more sense now and have no concerns over the integrity of the science conducted here. The article reads a lot clearer now so I am happy to recommend for publication now. Well done for your continued work in this important area or research.

I have noticed a few minor type-os:

Line 90 can you check the wording, I think 'having been' or 'being' should be substituted for 'been'.

Also use of square brackets where parentheses should be instead on line 77.

Many thanks.

**********

what does this mean?). If published, this will include your full peer review and any attached files.

If you choose “no”, your identity will remain anonymous but your review may still be made public.

**Do you want your identity to be public for this peer review?**  For information about this choice, including consent withdrawal, please see our Privacy Policy

Reviewer #1: No

**********

# Editor report: Acceptance letter

**DOI:** 10.1371/journal.pone.0351822.r004

**Authors:** Amitava Mukherjee

PONE-D-26-27071R1

PLOS One

Dear Dr. Unsworth,

I'm pleased to inform you that your manuscript has been deemed suitable for publication in PLOS One. Congratulations! Your manuscript is now being handed over to our production team.

At this stage, our production department will prepare your paper for publication. This includes ensuring the following:

* All references, tables, and figures are properly cited

* All relevant supporting information is included in the manuscript submission,

* There are no issues that prevent the paper from being properly typeset

You will receive further instructions from the production team, including instructions on how to review your proof when it is ready. Please keep in mind that we are working through a large volume of accepted articles, so please give us a few days to review your paper and let you know the next and final steps.

Lastly, if your institution or institutions have a press office, please let them know about your upcoming paper now to help maximize its impact. If they'll be preparing press materials, please inform our press team within the next 48 hours. Your manuscript will remain under strict press embargo until 2 pm Eastern Time on the date of publication. For more information, please contact onepress@plos.org.

You will receive an invoice from PLOS for your publication fee after your manuscript has reached the completed accept phase. If you receive an email requesting payment before acceptance or for any other service, this may be a phishing scheme. Learn how to identify phishing emails and protect your accounts at https://explore.plos.org/phishing.

If we can help with anything else, please email us at customercare@plos.org.

Thank you for submitting your work to PLOS ONE and supporting open access.

Kind regards,

PLOS ONE Editorial Office Staff

on behalf of

Professor Dr. Amitava Mukherjee

Academic Editor

PLOS One
